# Incidental and Asymptomatic Splenic Infarction and Infrarenal Thrombus in a COVID-19 Patient

**DOI:** 10.7759/cureus.26555

**Published:** 2022-07-04

**Authors:** Jordan Childers, Tuong Vi C Do, Forest Smith, Avinash Vangara, Subramanya Shyam Ganti, Ramya Akella

**Affiliations:** 1 Internal Medicine, Appalachian Regional Healthcare, Harlan, USA; 2 Internal Medicine, West Anaheim Medical Center, Anaheim, USA; 3 Internal Medicine/Pulmonary Critical Care, Appalachian Regional Healthcare Internal Medicine Residency Program, Harlan, USA; 4 Internal Medicine, Pikeville Medical Center, Pikeville, USA

**Keywords:** splenic thrombus, hypercoagulable state, infrarenal thrombus, splenic infarction, covid-19

## Abstract

The cytokine storm associated with coronavirus disease 2019 (COVID-19) triggers a hypercoagulable state leading to venous and arterial thromboembolism. Lab findings associated with this phenomenon are elevated D-dimer, fibrinogen, C-reactive protein (CRP), ferritin, and procalcitonin. We present the case of a 66-year-old male with dyslipidemia who was diagnosed with COVID-19 with worsening shortness of breath, myalgia, and loss of taste. Physical examination was remarkable for crackles with diminished lung sounds and use of his accessory muscles. Labs showed normal white blood cell count, D-dimer of 1.42 mg/L, ferritin of 961 ng/mL, lactate dehydrogenase (LDH) of 621 U/L, and CRP of 2.1 mg/dL. Chest X-ray showed atypical pneumonitis with patchy abnormalities. He required oxygen supplementation with fraction of inspired oxygen of 100% proning as tolerated. He received remdesivir, ceftriaxone, azithromycin, dexamethasone, prophylactic enoxaparin, and a unit of plasma therapy. His D-dimer had increased from 1.65 to 3.51 mg/L with worsening dyspnea. At this time, computed tomography angiogram (CTA) of the chest showed extensive ground-glass opacities and a 2.4 × 1.9 × 1.3 cm distal thoracic aortic intraluminal thrombus. He was started on a heparin drip. A follow-up CTA of the aorta showed thrombus or hypoattenuation within the splenic artery and wedge-shaped areas extending from the hilum with possible infarction and a 6 mm thrombus in the infrarenal abdominal aorta. He was transitioned to enoxaparin 1 mg/kg twice daily. He remained asymptomatic from his splenic infarction. This case adds more insight to splenic infarction associated with COVID-19 in addition to the 32 reported cases documented thus far. Management of thromboembolism includes a therapeutic dose of anticoagulation. To prevent thromboembolism, prophylactic anticoagulation is recommended for those hospitalized with COVID-19.

## Introduction

During the coronavirus disease 2019 (COVID-19) pandemic, the pathogenesis and complications were studied intently, including arterial and venous thromboembolisms, encephalopathy, barotrauma, and superimposed fungal infections [1-7]. COVID-19 pathogenesis involves excessive inflammation triggered from the viral infection due to profound hypoxia leading to diffuse intravascular coagulation. This triggers an associated cytokine storm which leads to a hypercoagulable state from Virchow’s triad of endothelial dysfunction, stasis, and platelet activation [2-5]. COVID-19 venous and arterial thromboembolisms include ischemic stroke, pulmonary embolism (PE), deep vein thrombosis (DVT), myocardial infarction, and systemic arterial embolism [6,7]. Studies have found an association between elevated D-dimer, fibrinogen, C-reactive protein (CRP), ferritin, and procalcitonin with COVID-19 thromboembolisms [8].

## Case presentation

A 66-year-old male with dyslipidemia presented 11 days after a diagnosis of COVID-19. He had worsening shortness of breath, myalgia, loss of taste, and feeling sick in general. He denied any diarrhea, nausea, or vomiting. On physical examination, he had crackles with diminished lung sounds using his accessory muscles to breathe. He was hypoxic with an oxygen saturation of 81% on room air. Labs showed normal white blood cell count, D-dimer of 1.42 mg/L, ferritin of 961 ng/mL, lactate dehydrogenase (LDH) of 621 U/L, and C-reactive protein (CRP) of 2.1 mg/dL. Chest X-ray showed atypical pneumonitis with patchy abnormalities. He was started on oxygen supplementation with settings on the vapotherm being 40 L with a fraction of inspired oxygen (FiO_2_) of 100% proning as tolerated. He received dexamethasone daily, prophylactic enoxaparin daily, a unit of plasma therapy, remdesivir, ceftriaxone, and azithromycin. On day two, as he had progressive hypoxemia with an elevated D-dimer of 2.64 mg/L, his enoxaparin was increased to twice daily dosage.

**Figure 1:**
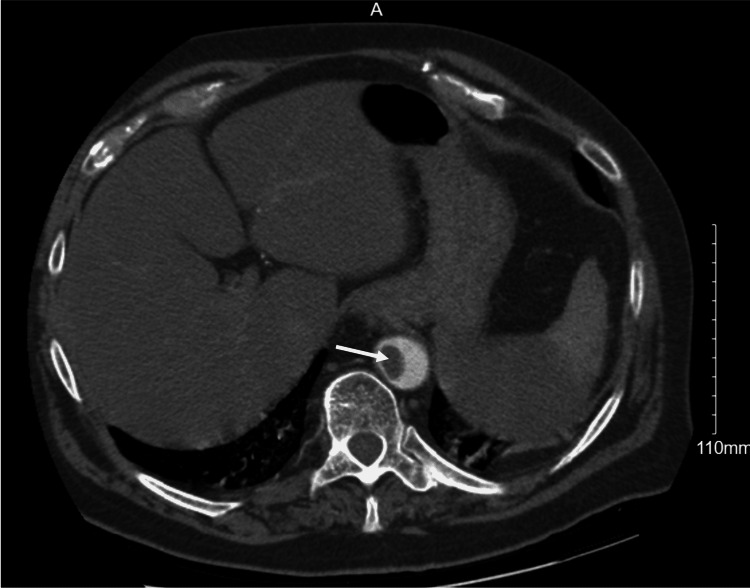
Computed tomography angiogram of the chest showing intraluminal thrombus in the distal thoracic aorta.

**Figure 2:**
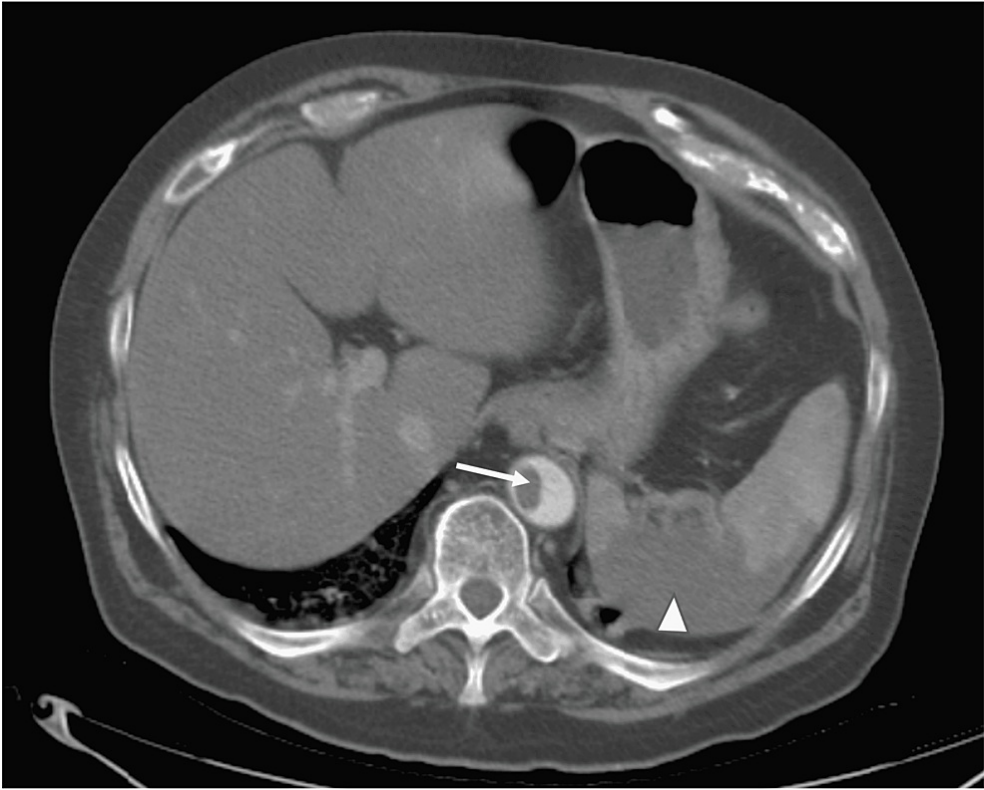
Computed tomography angiogram of the aorta showing thrombus within the splenic artery with wedge-shaped areas for possible infarction (arrowhead) and a 6 mm thrombus in the infrarenal abdominal aorta (arrow).

**Figure 3:**
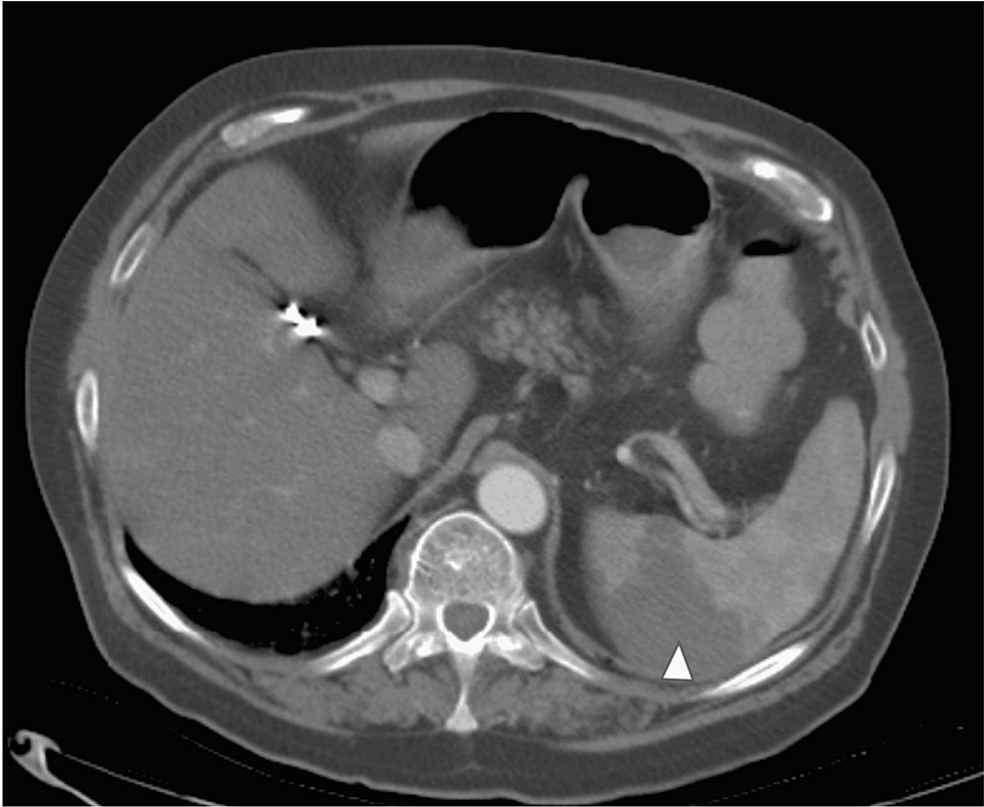
Computed tomography angiogram of the aorta showing hypoattenuation within the splenic artery for wedge-shaped areas around the hilum with possible infarction.

On day five, he required continuous positive airway pressure (CPAP) with pressure support of 10 and FiO_2_ of 85%. His D-dimer at this point increased from 1.65 to 3.51 mg/L. A computed tomography angiogram (CTA) of the chest was done which showed extensive ground-glass opacities with an incidental finding of a 2.4 × 1.9 × 1.3 cm intraluminal thrombus in the distal thoracic aorta (Figure 1). At this time, he was started on a heparin drip in the event that surgical intervention would be needed, and enoxaparin was discontinued. Further evaluation with a CTA of the aorta revealed a thrombus or hypoattenuation within the splenic artery, wedge-shaped areas around the hilum with possible infarction, and a 6 mm thrombus in the infrarenal abdominal aorta (Figures 2, 3). The vascular surgeon recommended no surgical intervention, so his heparin drip was discontinued two days later. He was then started on enoxaparin 1 mg/kg twice daily. His D-dimer peaked at 6.92 mg/L but then trended down. Unfortunately, the patient expired secondary to worsening hypoxemia but never developed any abdominal symptoms or limb ischemia.

## Discussion

COVID-19 hypercoagulable state has been well documented with an association between elevated D-dimer, fibrinogen, and interleukin (IL)-6 levels [8]. Our patient did have an elevated D-dimer which further increased secondary to the cytokine storm as his oxygenation status worsened. Klok et al. [6] found independent predictors of thrombotic complications as the mean age of 64 and coagulopathy of either prothrombin time of more than three seconds or activated partial thromboplastin time of more than five seconds [6]. Our patient’s age was 66 years. Kashi et al. [9] showed that a history of cardiovascular disease was linked to their cases of arterial thrombus events [9]. The patient’s age and dyslipidemia are risk factors for cardiovascular disease in this case. He had no history of any coronary artery disease or hypertension.

The most common thrombotic complications from COVID-19 infection are DVT and PE, with other systemic arteries such as the coronary arteries and radial artery being rare [2,5,6,10]. Our patient was found to have a thrombus in the thoracic aorta, splenic artery, and renal artery. No prior CT abdomen imaging was available for comparison. In the study by Klok et al. [6] of 184 patients with COVID-19 pneumonia, there was a 27% occurrence of venous thromboembolism and 3.7% arterial thrombotic events for a total incidence of 31% [6]. In the study by de Roquetaillade et al. [5], there were 20 out of 209 patients (9.6% incidence rate) with arterial thromboembolic events, with three patients having splenic infarcts [5]. Therefore, our case adds to the number of splenic infarctions seen in COVID-19 because it is not as common or is being underreported as most cases are asymptomatic and discovered incidentally.

We found 32 cases reporting similar complications of splenic infarction as a rare arterial thrombotic complication of COVID-19, as described in Table 1, as of April 2022 [2,5,7,11-31]. Most patients often report mild-to-moderate left-sided dull abdominal pain but can be asymptomatic without any abdominal pain [11,12]. Symptomatic arterial thrombosis is diagnosed clinically and on imaging with CTA, which is preferable [2,7,12]. Our patient’s splenic infarction was discovered incidentally on CTA as he was asymptomatic. There was no previous CTA of the abdomen per the patient and chart review to compare to. He was already receiving prophylactic anticoagulation which was adjusted to full therapeutic dosage once the thrombus was diagnosed. Further complications of splenic infarction include progression to lower limb ischemia, mobile aortic thrombi, pseudocyst formation, hemorrhage, splenic rupture, splenic aneurysm, or splenic abscess [9,16]. Our patient was monitored throughout his hospital course for any complications and never developed any lower limb ischemia. Unfortunately, our patient expired during his hospitalization.

**Table 1:** Cases of splenic infarction seen in those with COVID-19. F: female; sq: subcutaneous; TID: three times a day; LMWH: low-molecular-weight heparin; PFO: patent foramen ovale; M: male; HTN: hypertension; BID: twice a day; PE: pulmonary embolism; OSA: obstructive sleep apnea; IgG: immunoglobulin G; LDH: lactate dehydrogenase; GI: gastrointestinal; DM: diabetes mellitus; CKD: chronic kidney disease; CAD: coronary artery disease; HLD: hyperlipidemia; IV: intravenous; CRP: C-reactive protein; ACS: acute coronary syndrome

Study	Patient	Comorbidities	Symptoms	Complications	Labs	Treatment	Outcome
Mahmood et al. 2021 [2]	27, F	None	Abdominal pain	Possible splenic hemorrhage	D-dimer peak at >20 µg/mL	Prophylactic heparin 5,000 U sq TID daily transitioned to LMWH	Discharged on apixaban
de Roquetaillade et al. 2021 [5]	Three patients	Not reported besides 1 with PFO	Not reported	Not reported	Not reported	Not reported	Not reported
Dennison et al. 2021 [7]	70, M	HTN	Left lower quadrant abdominal pain	Bilateral rectus sheath hematomas, mesenteric vessel microhemorrhage	D-dimer 3.90 mg/mL	Prophylactic enoxaparin transitioned to LMWH 80 mg BID	Discharged
Santos et al. 2020 [11]	67, M	HTN	Asymptomatic	Pulmonary PE	Not reported	Not reported	Not reported
	53, F	None	Asymptomatic	None	Not reported	Not reported	Not reported
Qasim et al. 2020 [12]	60s, M	Asthma, OSA, morbid obesity, HTN, IgG deficiency	Dull left-sided abdominal pain	None	D dimer 1,088 ng/mL, ferritin 3,038 ng/mL	Prophylactic enoxaparin 40 mg BID transitioned to heparin drip for 24 hours, followed by enoxaparin 1 mg/kg BID	Discharged on rivaroxaban
Ramanathan et al. 2021 [13]	54, M	Obese	Sharp abdominal pain, nausea, vomiting	Kidney infarction	D-dimer 1.55 µg/mL, ferritin 1,633 ng/mL, LDH 2,136 U/L	Heparin drip at 18 U/kg/hour	Discharged on apixaban
Imoto et al. 2021 [14]	54, M	Not reported	Asymptomatic within GI	Multiple cerebral infarcts, bilateral renal infarcts	Not reported	Lovenox	Expired
Besutti et al. 2020 [15]	53, M	HTN, previous mitral valve replacement	Severe left flank pain	Left kidney infarct	Not reported	LWWH 6,000 U BID for 2 days	Discharged
	72, M	HTN, DM type 2, CKD stage 3, CAD	Severe abdominal pain	Small bowel ischemia	D-dimer 6,910 ng/mL	LMWH 4,000 U/day along with acetylsalicylic acid, resection of ischemic bowel, splenectomy, transitioned to heparin drip	Not reported
Sztajnbok et al. 2021 [16]	60, F	None	Asymptomatic	Aortic thrombosis of descending aorta	D-dimer 4,057 ng/mL, ferritin 719 ng/mL	Prophylactic LMWH 60 mg/day, transitioned to LMWH 60 mg BID	Discharged on warfarin
Hossri et al. 2020 [17]	29, F	Sickle cell disease	Abdominal pain, vomiting	Ischemic stroke	D-dimer 2,822 ng/mL, ferritin 4,511 ng/mL	Heparin drip	Not reported
Karki et al. 2020 [18]	32, M	None	Severe periumbilical pain	Splenic laceration with hemoperitoneum	Not reported	Supportive care	Not reported
Bradley et al. 2021 [19]	76, F	HLD	Not reported	Subarachnoid hemorrhages, pulmonary thrombus, myocarditis	Not reported	Not reported	Expired
Tranca et al. 2021 [20]	31, F	None	Mild dull abdominal pain	Not reported	Not reported	Enoxaparin 1 mg/kg BID, aspirin	Not reported
Rigual et al. 2021 [21]	53, M	Not reported	Not reported	Ischemic stroke, hemorrhagic splenic infarct, bilateral renal infarction, splenic pseudoaneurysm	Not reported	IV thrombolysis and mechanical thrombectomy, followed by enoxaparin 1 mg/kg daily, acetylsalicylic acid 100 mg	Discharged on acetylsalicylic acid
Ghalib et al. 2021 [22]	67, F	HTN, DM, CAD, asthma	Asymptomatic	None	D-dimer 1,072 ng/mL, ferritin 536 ug/L, CRP 163.3 mg/L	Therapeutic heparin infusion	Discharged on LMWH
Vidali et al. 2021 [23]	70, F	Not reported	Left upper quadrant pain	Thrombosis of extrahepatic and intrahepatic portal branches, thrombosis of splenic and mesenteric veins	CRP 10.8 mg/dL, LDH 248 U/L, D-dimer 4,926 ng/mL	LMWH 8,000 U	Not reported
Moradi et al. 2021 [24]	59, F	DM, HTN, HLD	Left upper quadrant pain, left flank pain	Limb ischemia	Normal D-dimer	Heparin drip	Discharged on rivaroxaban, aspirin, clopidogrel
Ceci et al. 2021 [25]	47, M	HLD, DM	Acute abdominal pain	ACS, myocarditis, kidney infarction	Not reported	Aspirin, clopidogrel, enoxaparin 1 mg/kg BID, then transitioned to unfractionated heparin drip; transitioned to warfarin; transitioned to enoxaparin 1 mg/kg BID	Discharged on warfarin
Abdelmohsen et al. 2021 [26]	Three patients	Not reported	Not reported	One had small bowel infarcts	Not reported	Not reported	Not reported
Mavraganis et al. 2022 [27]	64, M	None	Severe abdominal pain	Renal thrombosis, splenic vein thrombosis, thoracic aorta thrombi, renal infarct	LDH 1,244 U/L, D-dimer 3.7 mg/L, CRP 1.7 mg/dL	LMWH 6,000 U daily, then transitioned to enoxaparin 8,000 U BID with acetylsalicylic acid 80 mg daily, transitioned to fondaparinux sq 7.5 mg	Discharged on acetylsalicylic acid 80 mg and fondaparinux 7.5 mg sq
Berrichi et al. 2021 [28]	45, M	None	Acute abdominal pain in the left upper quadrant	Acute limb ischemia, renal infarcts, thrombosis of splenic vein	Not reported	IV unfractionated heparin 80 U/kg, thrombectomy, followed by unfractionated heparin drip at 18 U/kg/hour	Discharged
Javaid et al. 2022 [29]	44, M	HTN, obesity	Severe abdominal pain	None	Not reported	Supportive	Discharged
Rea et al. 2021 [30]	Three patients	Not reported	Not reported	Not reported	Not reported	Not reported	Not reported
Guillet et al. 2020 [31]	57, M	DM, obesity	Not reported	Mesenteric thrombi, renal infarction, lower limbs ischemia	D-dimer 1,169 µg/L, CRP 139 mg/L	Prophylactic LMWH transitioned to low-dose acetylsalicylic acid and IV unfractionated heparin	Discharged on warfarin

To prevent COVID-19 complications from its hypercoagulable state, the American Thoracic Society recommends that all hospitalized patients with COVID-19 receive thromboprophylaxis therapy of low-molecular-weight heparin (LMWH) or fondaparinux over unfractionated heparin and direct oral anticoagulants (DOACs) unless it is contraindicated [2,32]. LMWH is associated with lower mortality, as reported by Qasim et al. [12], with more benefits seen in those with severe COVID-19 or D-dimer greater than six times the upper limit of normal [12]. The concern with DOACs is their drug interactions with tocilizumab and the need to be renally dosed [3]. Kidney failure has been seen in those with COVID-19 due to patients being kept fluid-negative and as a side effect of remdesivir. For critically ill patients with proximal DVT or PE, it is recommended to use parenteral anticoagulation therapy with therapeutic weight-adjusted LMWH or fondaparinux over unfractionated heparin [4,14,32]. Our patient was already started on LMWH on admission with increased dosage as his D-dimer kept elevating. However, he was only transitioned to a heparin drip in case he needed surgical intervention because it has a shorter half-life of 45 minutes when compared to the half-life of four to five hours of LMWH.

For those who are ready for discharge, it is recommended that they be transitioned to DOACs or LMWH. Although arterial and venous thromboembolism can be treated with catheter-directed therapies, it is not an option for those with COVID-19 because it exposes healthcare workers to COVID-19 unless they are critically ill [4]. Bikdeli et al. [4] concluded that an inferior vena cava filter is indicated if there is recurrent PE despite anticoagulation or significant venous thromboembolism with absolute contraindications to anticoagulation.

## Conclusions

In patients hospitalized with COVID-19, a high index of suspicion is necessary for detecting thromboembolism, especially when they present with acute abdominal pain, chest pain, or lower extremity pain. Although PE is the most common thromboembolism complication of COVID-19, arterial thrombosis occurs in the coronary, abdominal, and even cranial arteries. Physicians need to consider arterial and venous thrombosis and investigate appropriately to manage it earlier in the course of the disease. Early detection will have better prognostic implications for patients given that prompt anticoagulation would reduce the risk of complications secondary to end-organ ischemia that results from thromboembolic events. Prophylactic anticoagulation is recommended for hospitalized COVID-19 with either LMWH or fondaparinux.
